# Holding the keys to health? A scoping study of the population health impacts of automated vehicles

**DOI:** 10.1186/s12889-019-7580-9

**Published:** 2019-09-11

**Authors:** Jennifer Dean, Alexander J. Wray, Lucas Braun, Jeffrey M. Casello, Lindsay McCallum, Stephanie Gower

**Affiliations:** 1 School of Planning, University of Waterloo, 200 University Avenue West, Waterloo, ON N2L 3G1 Canada; 2 Human Environments Analysis Lab, Department of Geography, Western University, London, ON Canada; 3 Department of Civil and Environmental Engineering, University of Waterloo, 200 University Ave, West, Waterloo, ON N2L 3G1 Canada; 4 Toronto Public Health, 277 Victoria St., 7th Floor, Toronto, ON M5B 1W2 Canada; 5 Dalla Lana School of Public Health, University of Toronto, 155 College St, Toronto, ON M5T 3M7 Canada

**Keywords:** Automated vehicles, Autonomous vehicles, Driverless cars, Public health, Road safety, Health equity, Built environment, Scoping review, Transportation planning, Mobility

## Abstract

**Background:**

Automated Vehicles (AVs) are central to the new mobility paradigm that promises to transform transportation systems and cities across the globe. To date, much of the research on AVs has focused on technological advancements with little emphasis on how this emerging technology will impact population-level health. This scoping study examines the potential health impacts of AVs based on the existing literature.

**Methods:**

Using Arksey and O’Malley’s scoping protocol, we searched academic and ‘grey’ literature to anticipate the effects of AVs on human health.

**Results:**

Our search captured 43 information sources that discussed a least one of the five thematic areas related to health. The bulk of the evidence is related to road safety (*n* = 37), followed by a relatively equal distribution between social equity (*n* = 24), environment (*n* = 22), lifestyle (*n* = 20), and built environment (*n* = 18) themes. There is general agreement that AVs will improve road safety overall, thus reducing injuries and fatalities from human errors in operating motorized vehicles. However, the relationships with air quality, physical activity, and stress, among other health factors may be more complex. The broader health implications of AVs will be dependent on how the technology is adopted in various transportation systems. Regulatory action will be a significant determinant of how AVs could affect health, as well as how AVs influence social and environmental determinants of health.

**Conclusion:**

To support researchers and practitioners considering the health implications of AVs, we provide a conceptual map of the direct and indirect linkages between AV use and health outcomes. It is important that stakeholders, including public health agencies work to ensure that population health outcomes and equitable distribution of health impacts are priority considerations as regulators develop their response to AVs. We recommend that public health and transportation officials actively monitor trends in AV introduction and adoption, regulators focus on protecting human health and safety in AV implementation, and researchers work to expand the body of evidence surrounding AVs and population health.

## Background

Over the past five years, there has been substantive and growing interest in how the new mobility paradigm of intelligent vehicles will change the future of both transportation systems and cities themselves. Central to these discussions is the role that the impending arrival of automated vehicles (AVs) will play in urban accessibility and connectivity as well as personal mobility [1–4]. Much of the work on AVs to date has focused on the technological advancements to speed their arrival in mainstream transportation [5] as well as debates around ownership [6–8], connectivity [9, 10] and data privacy [11]. While AVs as the ‘future of transportation’ have generated an enormous amount of attention from researchers and policy-makers, there is a paucity of knowledge about how this emerging technology will impact population-level health and wellbeing. Given the potentially significant changes to the global mobility paradigm, we undertook a scoping study of existing literature to explore the potential health impacts of AVs.

### Automated Vehicles

Automated Vehicles (AVs) are a rapidly emerging technology expected to overtake humans as the primary operators of motorized transport [12]. The Society of Automotive Engineers [13] has defined six levels of automation that describe a balance between human and computer inputs. Levels 0–2 include vehicle features that support the human driver, while Levels 3–5 involve automated driving features with limited human involvement. Level 0 requires full human operation with vehicle features providing warnings and momentary assistance (e.g., blind spot warning). Level 1 includes minimal vehicle support features that assist the driver in steering or braking capacities (e.g., lane centring or adaptive cruise control), while Level 2 includes vehicle features that support the driver in both steering and braking. Level 3 involves automated features in some conditions (e.g., route change to detour around traffic) but requires a human driver take control of the vehicle when requested. Level 4 includes more automated features with the ability to drive in some conditions and does not require a human operator to take over (e.g., local driverless taxis). Level 5, or full automation, a vehicle can drive everywhere in all conditions (e.g. no pedals or steering wheel installed). While the timeline for introducing AVs to market is uncertain, widespread adoption is predicted to occur within the next decade [4, 14, 15].

While AV technology is being applied to a wide variety of transportation sectors including air, rail, and sea, it is the road-based deployments that are expected to have the most direct impact on urban transportation systems and population health. This includes automation of both passenger and freight vehicles. The transformational capacity of AVs has been likened to the automobile at the turn of the twentieth century [16], which resulted in a host of social, environmental, economic, and health consequences.

### Health implications of transportation

The linkages between transportation and health are now widely known and are the focus of a robust area of research and policy-making. The United States Department of Transportation (USDOT) recognizes five pathways through which transportation affects human health: active transportation, road safety, air quality, connectivity, and equity [17].

The choice between active and passive modes of transportation is a key influencer of physical activity levels [18]. The various masses and speeds of vehicles in a transportation system expose participants to significant safety hazards, particularly in a collision [19].

Carbon-intensive travel modes impact air and water quality in ways that profoundly influence human health directly (e.g., respiratory health) and indirectly (e.g., climate change) [20, 21]. The design of transportation infrastructure and linkages between land use and urban design elements can influence access to health promoting or inhibiting aspects of the built environment (e.g., health care services, food outlets) [18, 20, 21]. Finally, ubiquitous access to low-cost, reliable transportation is recognized as a key determinant of social equity and provides much needed access to other upstream determinants of health (e.g., employment) [22].

As governments and agencies around the world strive to improve road safety through programs such as Vision Zero [23], ensure fair and affordable access to transportation through the Sustainable Development Goals [24], and promote walkable communities for all ages through the World Health Organization’s Age-Friendly Cities program [25], the link between transportation and health is a top political and practical issue. Addressing these priorities requires further investigation into the range of impacts of emerging transportation technologies. Yet, in the recent boom of research on AVs, there is very little literature examining the broader health impacts of this emerging technology despite the reality that fully automated vehicles are already being tested in cities around the globe.

To date, much of the social benefits rhetoric has focused on AVs potential to mitigate two health issues. First, AVs will likely significantly improve road safety by reducing the number of automobile crashes caused by human error [1, 26, 27]. Further, AVs have been described as potentially improving independent mobility for populations who are unable to or can no longer drive (e.g., older adults) [1–4]. While important, these two issues do not include the broad range of potential impacts, both positive and negative, that AVs may have on health [28].

The bulk of the current literature on societal impacts of AVs is speculative, given the lack of empirical data on their operations. However, even testing phases have had health implications such as a widely publicized incident in March 2018 when a person was struck and killed by an Uber-owned AV in Arizona [29]. Moreover, emergent research suggests that public readiness for AVs is low. Researchers in Australia have begun to assess public awareness of AVs and found in a survey of over 1600 residents that there were low levels of awareness of the potential health benefits, with only 21% acknowledging a potential improvement in road safety [30]. Even in the short term, there is a clear need to assess the health impacts of adopting AV technology.

In this paper, we synthesize the current expectations of the impacts of AV technology, based on the known linkages between transportation and health. We then propose a conceptual map of and describe the most likely health outcomes from various implementation scenarios. We conclude by identifying the critical indicators that should be monitored by practitioners as AV adoption progresses; providing a set of ideal constraints on AV implementation to protect human health; and suggesting areas for further research or professional inquiry.

## Methods

**Fig. 1:**
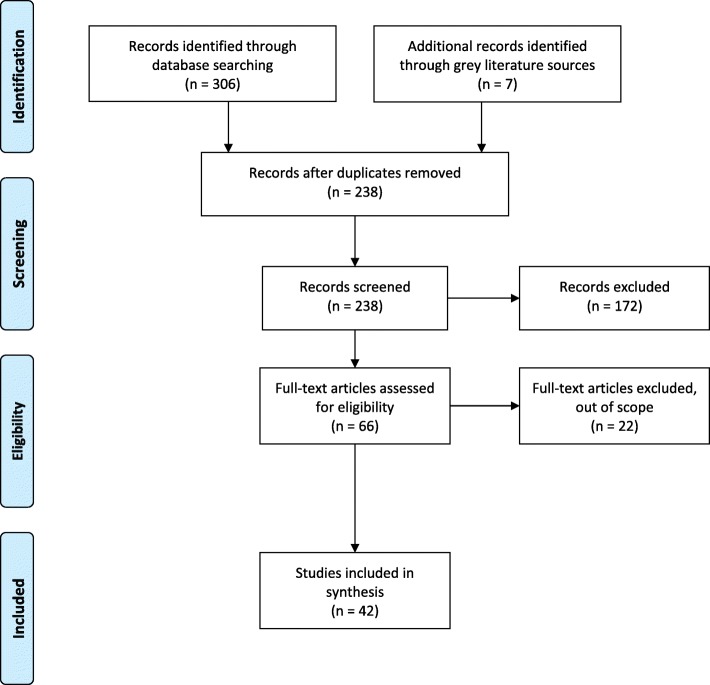
PRISMA diagram for reporting search results

The existing body of knowledge on AVs is broad and rapidly evolving. Accordingly, we adopt Arksey and O’Malley’s [31] scoping review protocol to assess the existing academic and grey literature for common themes, key findings, and knowledge gaps. Scoping reviews have certain advantages over traditional literature reviews, as the method allows for a systematic search of a rapidly evolving evidence base [32]. The method has been widely adopted in medicine and public health; with it becoming more popular in the social and natural sciences [32–34]. Wherever possible, we followed the PRISMA standard [35] for reporting items contained in the review (Fig. 1).

Our review is guided by the following question: what implications could AVs have for human health, based on the current understanding and state of knowledge about AV technology? We proceed with describing the search strategy, screening criteria, and coding approach used to synthesize the evidence.

### Search strategy

**Fig. 2:**
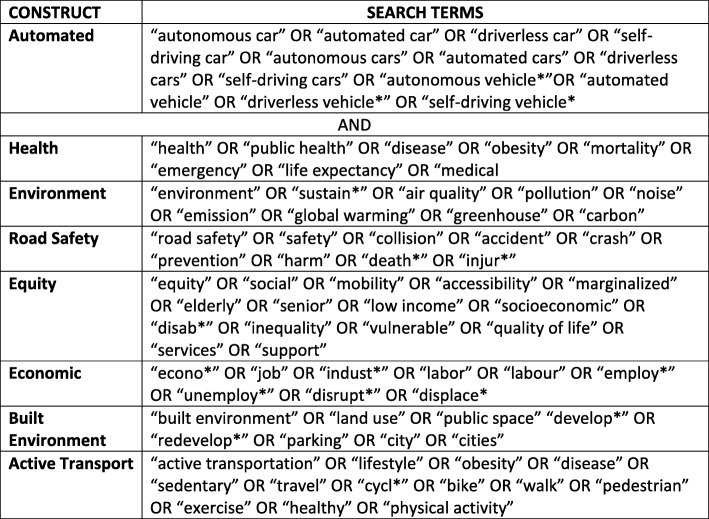
Search terms used to locate academic and grey literature

The search strategy consists of exploring academic and ‘grey’ literature repositories for evidence pertaining to the health impacts of AVs. We investigated a broad range of interdisciplinary databases, including: Canadian Research Index, Embase, GeoScan, PsycINFO, PubMed, Scopus, TRID, and Web of Science. The first search of automated vehicles and related terms returned tens of thousands of potential sources. The search strategy was then scoped in by adding in terms related to health, equity, road safety, mode choice, built form, environment, and synonyms to narrow the results to items relating to population health and transportation (Fig. 2). The first search was conducted in November 2017, while an update to the search was performed in December 2018.

### Screening criteria

Articles captured in the search were screened for relevance to the research question. Multiple raters reviewed the titles, abstracts, and full-texts of each result in a progressive fashion to arrive at the final dataset for synthesis. Articles were only included if they were published in English, addressed a specific determinant of health or wellbeing, and offered a rigorous evidence-based approach to their investigation. Duplicates of articles across databases were removed at the title screening stage. Disagreements about screening decisions were resolved by consensus.

### Coding and synthesis

Included results were processed by multiple raters using a piloted data extraction process. Coding items included bibliographic information, health impacts, and findings of each result. The health impacts were structured into five themes for coding purposes: road safety, natural environment, social equity, lifestyle, and built environment, which were closely aligned with the USDOT framework for health impacts from transportation [17]. Within each theme there were multiple subthemes that emerged from the data as relevant to AVs and health. Disagreements about coding decisions during this analysis were resolved by consensus.

## Results

The refined search terms returned over three hundred potential sources relevant to the research question. The scanning and screening process resulted in 43 items that are included in the synthesis (Table 1). The bulk of the evidence is related to road safety (*n* = 37), followed by a relatively equal distribution between social equity (*n* = 24), environment (*n* = 22), lifestyle (*n* = 20), and built environment (*n* = 18) themes. Each source was coded into multiple a priori themes and emergent subthemes, resulting in overlap between elements of the synthesis.

**Table 1:** Results organized by themes and subthemes

Road Safety (Theme)	
Collision Avoidance (Subtheme)	Altarum Institute [36], Arief et al. [37], Bahamonde-Birke et al. [38], Brown et al. [9], Cohen et al. [39], Conference Board of Canada [40], Crayton & Meier [1], Curl et al. [41], de Sio [42], Duarte & Ratti [43], Fagnant & Kockelman [14], Fleetwood [26], Freedman et al. [44], Harper [45], Harrow et al. [46], Kelley et al. [3], Litman [15], Luttrell [26], Marotte & Dixon [47], Menon et al. [48], Milakis et al. [4], Milakis et al. [49], Millard-Ball [50], Mladenovic et al. [7], Payre et al. [51], Pettigrew [2], Pettigrew et al. [29], RAND [10], Rodriguez et al. [52], Ryerson et al. [53], Sessa et al. [8], Thomopoulos & Givoni [6], Ticoll [54], Watkins [55], van Schalwyk & Mindell [28], Yeomans [56],
Transition Period	Altarum Institute [36], Bahamonde-Birke et al. [38], Brown et al. [9], Cohen et al. [39], Fitt et al. [41], Marotte & Dixon [47], Thomopoulos & Givoni [6]
Safety Testing	Altarum Institute [36], Arief et al. [37], Fleetwood [26], Kelley et al. [3], RAND [10], Yeomans [56]
Ethics	de Sio [42], Fleetwood [26], Harrow et al. [46], Rodriguez et al. [52], Ryerson et al. [53]
Hacking	Litman [15], Milakis et al. [4], Yeomans [56]
Organ Transplants	van Schalwyk & Mindell [28]
Social Equity	
Accessible Mobility	Altarum Institute [36], Bahamonde-Birke et al. [38], Brown et al. [9], Conference Board of Canada [40], Crayton & Meier [1], Fagnant & Kockelman [14], Fitt et al. [43], Harper et al. [43], Kelley [3], Litman [15], Marotte & Dixon [47], Milakis et al. [4], Milakis et al. [49], Mladenovic et al. [7], Pettigrew [2], Pettigrew et al. [29], RAND [10], Thomopoulos & Givoni [6], Ticoll [54], van Schalkwyk & Mindell [28], Sessa et al. [8], Watkins [55]
Employment	Altarum Institute [36], Clements & Kockelman [57], Conference Board of Canada [40], Marotte & Dixon [47], Milakis et al. [4], Milakis et al. [49]
Affordable Housing	Milakis et al. [4]
Natural Environment	
Fuel Efficiency and Emissions	Altarum Institute [36], Amberg [58], Bahamonde-Birke et al. [38], Brown et al. [9], Cohen et al. [39], Conference Board of Canada [40], Crayton & Meier [1], Fagnant & Kockelman [14], Fitt et al. [41], Altshuler at al. [59], Harrow et al. [46], Marotte & Dixon [47], Merlin [60], Milakis et al. [4], Pettigrew [2], Pettigrew et al. [29], RAND [10], Sessa et al. [8], Tomopoulos & Givoni [6], van Schalkwyk & Mindell [28], Wadud et al. [12]
Vehicle Kilometres Travelled	Amberg [57], Brown et al. [9], CPHA [40], Cohena et al. [38], Crayton & Meier [1], Fagnant & Kockelman [14], Harrow et al. [46], Merlin [60], Milakis et al. [4], Sessa et al. [8], Wadud et al. [12]
Life Cycle	Menon et al. [48], Milakis et al. [4], Pettigrew [2]
Noise	Milakis et al. [4], RAND [10]
Lifestyles	
Mode Choice	Bahamonde-Birke et al. [38], Crayton & Meier [1], Milakis et al. [4], Marotte & Dixon [47], Merlin [60], Sessa et al. [8], Thomopoulos & Givoni [6], Ticoll [54], van Schalkwyk & Mindell [27], Watkins [55], Yap et al. [61]
Travel Enjoyment	Altarum Institute [36], Bahamonde-Birke et al. [38], Crayton & Meier [1], Curl et al. [41], Fagnant & Kockelman [14], Harrow et al. [46], Litman [15], Morris & Guerra [62], Pettigrew et al. [29], Thomopoulos & Givoni [6]
Public Perception	Harrow et al. [46], Menon et al. [48], Payre et al. [51]
Built Environment	
Land Use and Transportation	Altarum Institute [36], Bahamonde-Birke et al. [38], Brown et al. [9], Clements & Kockelman [57], Crayton & Meier [1], Conference Board of Canada [40], Duarte & Ratti [43], Fitt et al. [41], Fagnant & Kockelman [14], Harrow et al. [46], Milakis et al. [4], Milakis et al. [49], Mladenovic et al. [7], RAND [10], Sessa et al. [8], Thomopoulos & Givoni [6], Ticoll [54], Watkins [55]

Note: Items may be coded into multiple themes and subthemes

### Road safety

There are 37 items related to road safety, with 27 from academic sources and 10 from grey sources. Most evidence in this theme is related to collision avoidance (*n* = 27), with less relating to transition period (*n* = 7), safety testing the technology (*n* = 6), ethics (*n* = 5), hacking (*n* = 3), and organ transplants (*n* = 1). AVs are predominantly viewed as a positive improvement to current road safety conditions as they eliminate human error, the most common reason for collisions [1, 2, 9, 27, 40]. However, improvements in road safety from autonomous vehicles would likely reduce the availability of human organs for transplant [2].

### Social equity

There are 24 items related to social equity, with 16 from academic sources and eight from grey sources. Accessible mobility (*n* = 23) is of primary concern in this theme with some discussion of employment impacts (*n* = 6), and affordable housing (*n* = 1). AVs are predicted to improve accessibility for differently abled populations, reduce isolation, and improve social connectivity [1, 3, 40]. However, AVs could further perpetuate existing societal inequalities in the transportation system depending on the ownership and access models (i.e., shared vs personal ownership) [6, 36]. For example, if private ownership of AVs is the prevailing model, high income populations will be more likely to benefit from on-demand mobility while lower income populations may face decreased access to transportation, longer commute times or limited accessibility to desirable destinations by relying on public transportation.

### Natural environment

There are 22 items related to the natural environment, with 17 from academic sources and five from grey sources. The evidence base is predominantly interested in the fuel efficiency or emissions of AVs (*n* = 21), and implications for vehicle kilometres travelled (*n* = 11). There is also minor interest in the life cycle of AVs (*n* = 3), and noise impacts (*n* = 2). Authors are split on the implications of AVs, with most arguing they will render a more efficient and less carbon-intensive transportation system if the majority of AVs are electrically powered [4, 6, 10]. Others argue that AVs will further exacerbate existing automobile dependency requiring more road infrastructure with deleterious impacts to the natural environment [8, 9, 14, 47]. Again, the model of AV ownership and access, coupled with the type of fuel source, will determine the actual environmental impacts.

### Lifestyle

There are 20 items related to lifestyles, with 14 from academic sources and six from grey sources. Changes in travel mode choice (*n* = 11) and the effects of travel by AVs on overall travel enjoyment or stress (*n* = 10) are interdependent areas of interest in this theme. There is also limited research on public perceptions towards AV technology with evidence suggesting a lack of awareness of both their environmental and human health impacts (*n* = 3). AVs are expected to rapidly improve the user experience with private transport by removing the stressful element of driving the vehicle and limiting the amount of time spent in congested traffic conditions [1, 4, 6, 36, 62]. However, reliance on AVs could result in more sedentary behaviour as users opt to take AVs for trips that traditionally involved some form of active travel or rely on AVs for longer trips where they previously relied on rail or air travel [6, 8, 40]. The limits placed on AV use, and where they can operate will shape their influence on lifestyle factors.

### Built environment

There are 18 items related to the built environment, with 12 from academic sources and 6 from grey sources. All the evidence in this theme was related to the interactions between transportation systems and land use patterns (*n* = 18), and how use of AVs could affect the demand for parking, urban design, density, and distribution of right-of-way space between modes [1, 4, 6–10, 14, 40, 57]. The modification of land use and transport cycles in urbanized areas could have significant health implications. For example, the traffic efficiency of AVs could free up space in the right-of-way to allow for cycling infrastructure and wider sidewalks for pedestrians. Further, a shared model of AVs would allow for reclaiming parking lots as part of the public realm and present opportunities for affordable housing or urban green spaces [1].

## Discussion

The existing literature suggests that AVs, like other modes of transportation, have a clear and direct role in shaping the social and environmental determinants of health. There was almost universal agreement in the literature that automated driving will result in significantly fewer road-related injuries and fatalities. However, this reliance on technology rather than human operation of road vehicles comes with additional risks, such as power grid-failures and cyber-terrorism. Beyond road safety, there is a lack of consensus on the impacts of AVs on human health.

Much of the speculation and uncertainty is based on how AVs will be introduced into existing urban transport systems with the most important variables being ownership models, fuel sources, government regulations, and user experiences. For example, a fully shared and electrically-powered fleet of ultra-lightweight AVs that is fully integrated into an active transportation network will promote travel behaviours, urban forms, and climatic conditions that will contribute to improved health outcomes. A future with large individually-owned AVs powered by fossil fuels and operating with low occupancy is likely to decrease physical activity, degrade the climate, and exacerbate existing inequities, leading to poor health outcomes. Realistically, between these two extreme scenarios there exists a continuum of possible outcomes. Given the breadth of scenarios, we propose a ‘conceptual map’ that could be used to evaluate the potential health outcomes related to an element of AV implementation. We follow the discussion of this conceptual framework with recommendations for transportation planners, urban planners, public health practitioners, regulators, and researchers.

### The conceptual map of AV impacts on population health

**Fig. 3:**
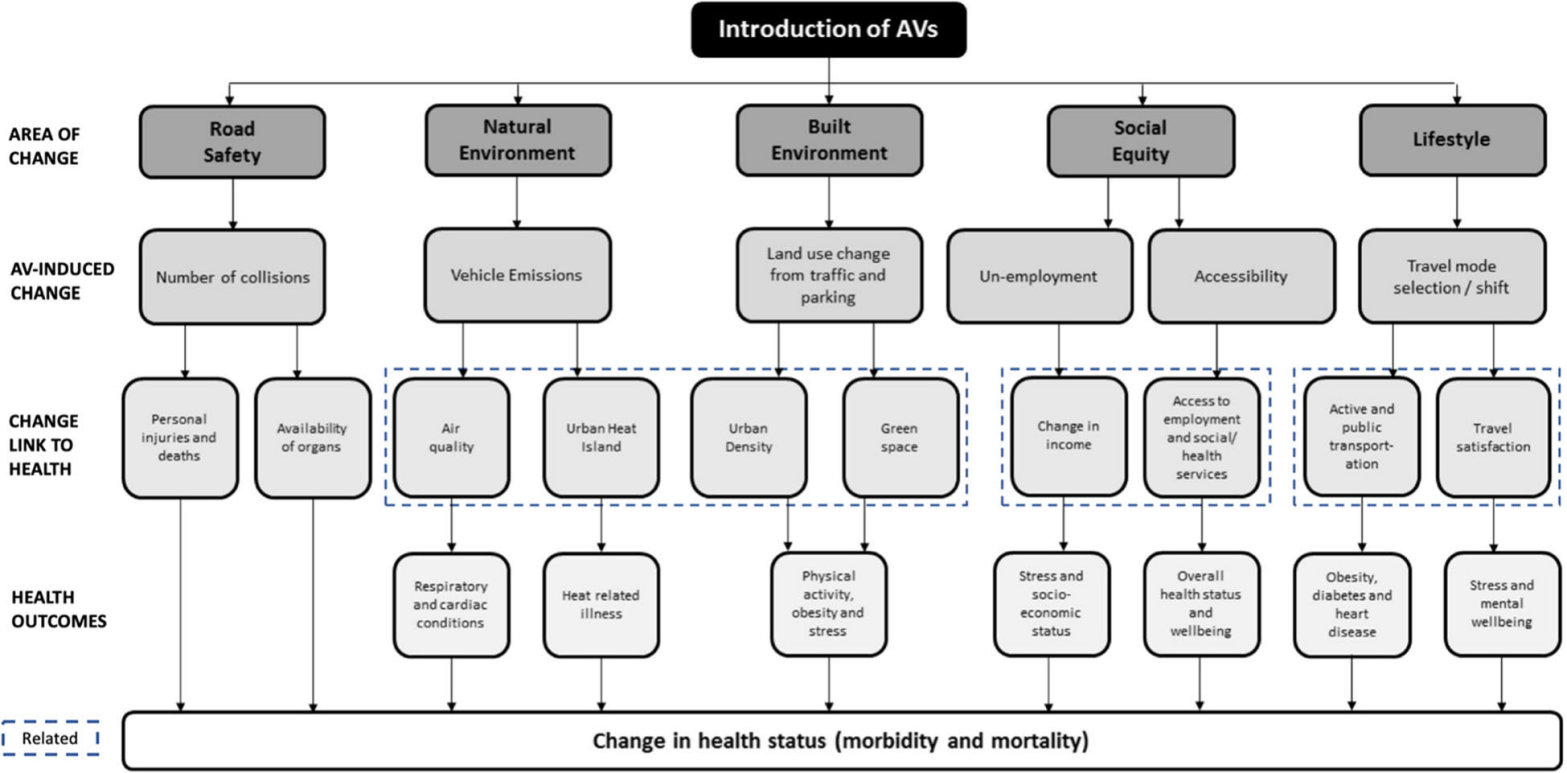
Conceptual framework for the linkages between AVs and health outcomes

Automated vehicles could have a range of direct and indirect health impacts. We structure these impacts as a pathway model of cause-effect dependencies (Fig. 3). These types of models are often used in the health and transport fields. For example, the Health Impact Pyramid [63] or Multi-Level Biological and Social Integrative Construct [64] provide structured models of nested intervention levels, and cause-and-effect pathways between an environmental factor and health outcomes [63, 64]. From a transportation perspective, modelling of cause-effect relationships has been an integral part of estimating changes to modality, as well as the health effects of transportation systems [65]. The construction of an interdisciplinary theoretical model that brings together disparate health and transportation related evidence can provide a unifying framework for all fields that are interested in AVs to engage in well-informed dialogues. Therefore, the intent of this figure is to capture our findings in an easily interpretable model of the cause-effect relationship between AVs and human health outcomes.

We begin at the top of the ‘map’ by outlining the thematic areas of change from the introduction of AVs (Fig. 3). These themes provide a structured set of impacts across the built and social environments that are associated with health. We then describe the specific change(s) that could occur, given the results of our review, in these thematic areas from greater adoption of AVs. We follow by providing the built, social or health effect dependencies of the AV-induced cause, noting interdependencies between the relationships. These links then lead to a specific health outcome, and/or a change in overall population morbidity and mortality from the chain of environmental health impacts. Thus, the purpose is to provide a navigational tool to explain the complex, and sometimes contradictory, health outcomes of AVs. For example, applying the map illustrates that our review found evidence that AVs could reduce motor vehicle collision. While this is a positive outcome in terms of public health, there is a potential ‘cost’ associated with this result; with fewer organs being made available for people who need a transplant.

Both the concept map and the body of literature it was developed from, predominantly focus on AVs in an urban context. However, there are likely differential health impacts related to road safety and equity for AVs in rural environments to reduced access to emergency services in the event of a collision. Overall, rurality is an underexplored area of AV research.

### Recommendations

There are complex relationships between AVs, social and environmental determinants, and health outcomes. Transportation, planning, and health practitioners should focus on the long-term objective of achieving the best possible health outcomes when considering the response to AV adoption including maintaining a focus on health equity. The impact of AVs on population health can be moderated by monitoring trends, prioritizing health in regulatory actions, incentivizing health and safety in vehicle design, and closing knowledge gaps.

#### Monitor trends

Most of the articles from public health authors stress the importance of actively monitoring AVs as they are introduced into the transportation system [2, 26, 35]. These authors advise that only by keeping up with the rapid progression of this technology can practitioners participate in discussions of regulation so that safety is prioritized and plan initiatives that equitably and proactively manage the impacts of AVs rather than provide a delayed response to their effects. A key factor to monitor will be safety performance in both simulated and live traffic conditions. Otherwise, the arrival of AVs before adequate testing is completed could pose a significant threat to road user safety.

#### Prioritize health

The literature suggests AVs could have a net benefit for human health and wellbeing. Researchers anticipate reductions in road fatalities and injuries, and improvements in overall social connectivity [2, 26, 35, 41]. However, there remains significant uncertainty with respect to several aspects of AV emergence, including the rate of adoption, technological capabilities, and ownership models. Without limits on use and discouragement of sole private ownership, there is a significant chance of decreased utilitarian physical activity, and inequities in accessibility and commute time for low-income populations. Thoughtful planning and community design is needed to integrate AVs into transportation networks in a way that supports safe and healthy travel for all modes; complementary policies may be able to promote affordability and encourage physical activity. Therefore, regulators and policymakers should adopt a precautionary approach that promotes the protection of public health and safety during the implementation of AVs in the transportation system. Our conceptual framework could be the ideal tool for use in scenario analyses to inform regulatory action.

#### Incentivize healthy and safe vehicle design

There are opportunities for AV manufacturers and developers to assist in the promotion of health by designing and programming vehicles to benefit road safety, equity, and natural environment. For instance, manufacturing vehicles that are electrically powered, and kept as light as possible will reduce energy consumption, reduce emission of harmful air pollutants, and benefit the natural environment. Further, assessing how to reconfigure seating arrangements inside an AV will better protect passengers (who do not need to interact with vehicle controls or observe their surroundings) in the event of a collision. Governments can also provide incentives for manufacturers with the highest safety ratings and features. From a programming perspective, AVs will need to be capable of determining the best course of action (e.g., whether to collide with an individual or inanimate object) when a collision is inevitable, and manufacturers can be transparent about how these decisions are made and how they consider both road safety and equity. This has complex and distinct implications related to equity and road safety. Further, AVs may be programmed to connect with infrastructure and/or other vehicles on the road in order to increase road safety for all road users. There are also potential opportunities for governments to incentivize research and development on AV related software programs that will help promote healthy and safe design.

#### Close gaps

Most of literature, while relevant to health and wellbeing, was not written by authors with health credentials and affiliations. This finding stresses the importance of further engagement by the population health community in AV research. We suggest areas for further inquiry include: (1) the interaction between AVs and other road users such as cyclists, pedestrians and other micro-mobility users, as well as integration with public transit systems; (2) the broader land-use impacts of AVs in dense urban areas; (3) regulatory policies that promote population health through advantageous AV ownership models, fuel sources, and right of way; and, (4) identifying ways to measure and monitor health and equity impacts of AV adoption.

In 2018, global consulting firm KPMG began an annual ranking of countries’ readiness for autonomous vehicles based on several indicators including policy and legislative support for AV adoption [66]. Given the multiple linkages between AVs and population health impacts found in this review, it is important that public health stakeholders are included in policy-making decisions on AVs in order to ensure consideration of health impacts associated with the technology and facilitate healthy and safe integration of AVs on our roads.

## Conclusion

We have found that AVs are indeed a complex and multi-faceted population health and wellbeing issue. There are direct effects such as rates of road injury or vehicle emissions, and less obvious effects such as changes to urban form or commuting stress, which can ultimately affect health and wellbeing. The list of impacts found here is not exhaustive, as the link between AVs and health is a subject that requires further investigation. When discussing these potential impacts, it is equally important to consider the uncertainty and range of outcomes that exist. There are many factors that will influence the future of AVs, including the ownership model, the fuel source, and the corresponding regulation by public and private entities. The impacts that are experienced, and the degree to which they are felt depends entirely on which of these scenarios become reality. However, regulators are not passive participants in this paradigm shift. Action, or the lack thereof, will determine the role AVs assume in local, regional and global transportation systems. Sinasic and Wray [67] hypothesize that the degree of AV regulation will be the prime factor determining the economic, social, environmental, and health impacts of AVs. It is therefore crucial that all stakeholders, including public health agencies work to ensure that population health outcomes and equitable distribution of health impacts are priority considerations as regulators develop their response to AVs. In conclusion, we suggest that public health and transportation officials actively monitor trends in AV introduction and adoption, regulators focus on protecting human health and safety in AV implementation, and researchers work to expand the body of evidence surrounding AVs and population health.

## Data Availability

All data generated or analysed during this study are included in this published article.

## Abbreviations

AV: Automated Vehicle
PRISMA: Preferred Reporting Items for Systematic Reviews and Meta-Analyses
TRID: Transportation Research International Documentation
USDOT: United States Department of Transportation
